# Two experimental methods to integrate intra-oral scans into 3D stereophotogrammetric facial images

**DOI:** 10.1007/s00784-024-06138-8

**Published:** 2025-01-09

**Authors:** Reinout R.P. Schobben, Frits A. Rangel, Robin Bruggink, Marjolein L.D. Crins - de Koning, Ewald M. Bronkhorst, Edwin M. Ongkosuwito

**Affiliations:** 1 Department of Dentistry Section Orthodontics and Craniofacial Biology, Radboud University Medical Center, P.O. Box 9101, Nijmegen, 6500 HB The Netherlands; 2 Radboud University Medical Center, Radboudumc 3DLab, Nijmegen, The Netherlands; 3 Department of Dentistry, Radboud University Medical Center, Radboud Institute for Health Sciences, Nijmegen, The Netherlands

**Keywords:** Orthodontics, 3D stereophotogrammetry, Facial image, Intra-oral scan, CBCT, Integration

## Abstract

**Objectives:**

For this research two different ways for integrating intra-oral scans into three-dimensional (3D) stereophotogrammetric images are analyzed and compared to the gold standard method.

**Materials and methods:**

A cross-sectional study was performed. For each patient a complete dataset was collected, which was used to generate 3D fusion models by three different methods: method A using cheek retractors, method B using a tracer and method C using full-skull CBCT. The experimental methods A and B were compared to the gold standard method C.

**Results:**

A group of eighteen patients were included in this study. The translation (X, Y,Z), the euclidean distance and the rotation (roll, pitch, yaw) were calculated for both experimental methods A and B in comparison with the gold standard method C. Twelve out of fourteen measurements were clinically acceptable (below 2 mm or 2 degrees). Method A shows the highest deviation in the pitch-orientation for rotation (2.51 degrees, 95% CI [1.756 … 3.272]), while method B shows a higher deviation along the y-axis (1.85 mm, 95% CI [1.224 … 2.467]).

**Conclusions:**

This study shows promising results of non-ionizing methods to integrate intra-oral scans into 3D stereophotogrammetric images. With improved accuracy in pitch in method A and translation along the Y-axis in method B, all measurements will be within the clinically acceptable threshold. However, since these two measurements exceed the clinically acceptable thresholds, the complete model positioning is less accurate. Therefore the main goal in further research should be to improve the accuracy of the pitch in method A and the translation along the Y-axis in method B. Additionally, for clinical use the biggest improvement could be gained by optimizing the clinical workflow and data processing.

**Clinical relevance:**

By using a non-ionizing 3D fusion model instead of a conventional cephalogram for treatment planning, the ionizing dose during orthodontic treatment can be significantly reduced.

## Introduction

A cephalometric analysis based on placement of landmarks and tracing of cephalometric radiographs is part of orthodontic diagnosis and treatment planning since the 1930s [1]. A major disadvantage of cephalometric radiographs is that a three-dimensional structure is represented in a two-dimensional way [2–4]. Furthermore, patients are exposed to ionizing radiation to produce the cephalometric radiograph and this may be disadvantageous [5]. In concordance with the ALARA principle (As Low As Reasonably Achievable), the clinician should determine whether taking the cephalometric radiograph is justifiable and necessary for orthodontic diagnosis and treatment planning [6]. 

Existing literature suggests that cephalometric radiography may not be justifiable for patients with mild crowding or spacing, nor for treatment plans that do not change the maxillomandibular relationship [7]. Several studies have proven that there is no difference in orthodontic treatment planning with or without cephalometric information in Class II division 1 malocclusions or borderline extraction cases [8–11]. This makes justification for the standard use of ionizing radiation in orthodontic diagnosis and treatment planning in this era questionable.

A non-ionizing method for facial diagnosis and orthodontic treatment planning, is three-dimensional (3D) stereophotogrammetric imaging. 3D stereophotogrammetric scanners render reliable, accurate and reproducible images of the facial extra-oral soft tissues [12, 13]. However, a detailed display of a digital intra-oral structure can only be accurately acquired by intra-oral 3D scanners [14] or digitalization of conventional plaster casts.

The challenge is to create a fast and easy workflow to accurately integrate the intra-oral 3D images with the 3D extra-oral images to obtain a full and detailed 3D reconstruction of the face and oral cavity. Visualization of the relation between the intra- and extra-oral structures is not only helpful for orthodontic diagnostics and treatment planning, but also facilitates the evaluation of the relationship between the intra- and extra-oral morphology [15, 16]. A first attempt at this was already described by Van Loon et al. in 1915 with combining plaster dental casts and plaster casts of the face [17]. Nowadays, with the development of digital 3D technology, a complete digital workflow is available to create a 3D model of the face and dentition, fusing images of extra-oral soft tissues and intra-oral structures [18–20].

However, to create such a fusion model, Cone-Beam Computed Tomography (CBCT) images are necessary. By superimposing a 3D stereophotogrammetric extra-oral image on a soft tissue model obtained from a CBCT image [21] and by superimposing the 3D intra-oral image on the teeth in the CBCT image, the 3D fusion model can be made. This 3D fusion model provides a precise 3D mesh and photorealistic digital 3D representation of a patient’s face and intra-oral structures. This procedure is considered to be the gold standard from a technical point of view [22, 23]. However, a CBCT image requires the use of ionizing radiation. Therefore, a method that leads to the fusion of extra- and intra-oral 3D images without the use of CBCT would be beneficial for the patient.

Several studies have tried to find an accurate and reproducible way for the integration of digitalized dental casts directly into the 3D stereophotogrammetric image without using CBCT [19, 24–26]. Methods vary from using cheek retractors when capturing the 3D stereophotogrammetric image and superimposition on the visible anterior teeth, to the use of an extra-oral tracer which is attached to a bite plane when capturing the 3D stereophotogrammetric image.

Most of these studies however lack validation of the proposed method, i.e. the new method was not compared to the gold standard of superimposing on CBCT images. Furthermore, in none of these studies a direct comparison between the use of cheek retractors or extra-oral tracers was made. Finally, all of these studies used digitalized conventional plaster casts instead of intra-oral scans, where the use of intra-oral scans is the standard these days [27]. Our study will overcome these shortcomings and therefore aims to compare two different methods of integrating intra-oral scans in 3D stereophotogrammetric extra-oral images, and to compare those to the gold standard of superimposing on CBCT images.

## Materials & methods

### Study design

**Fig. 1:**
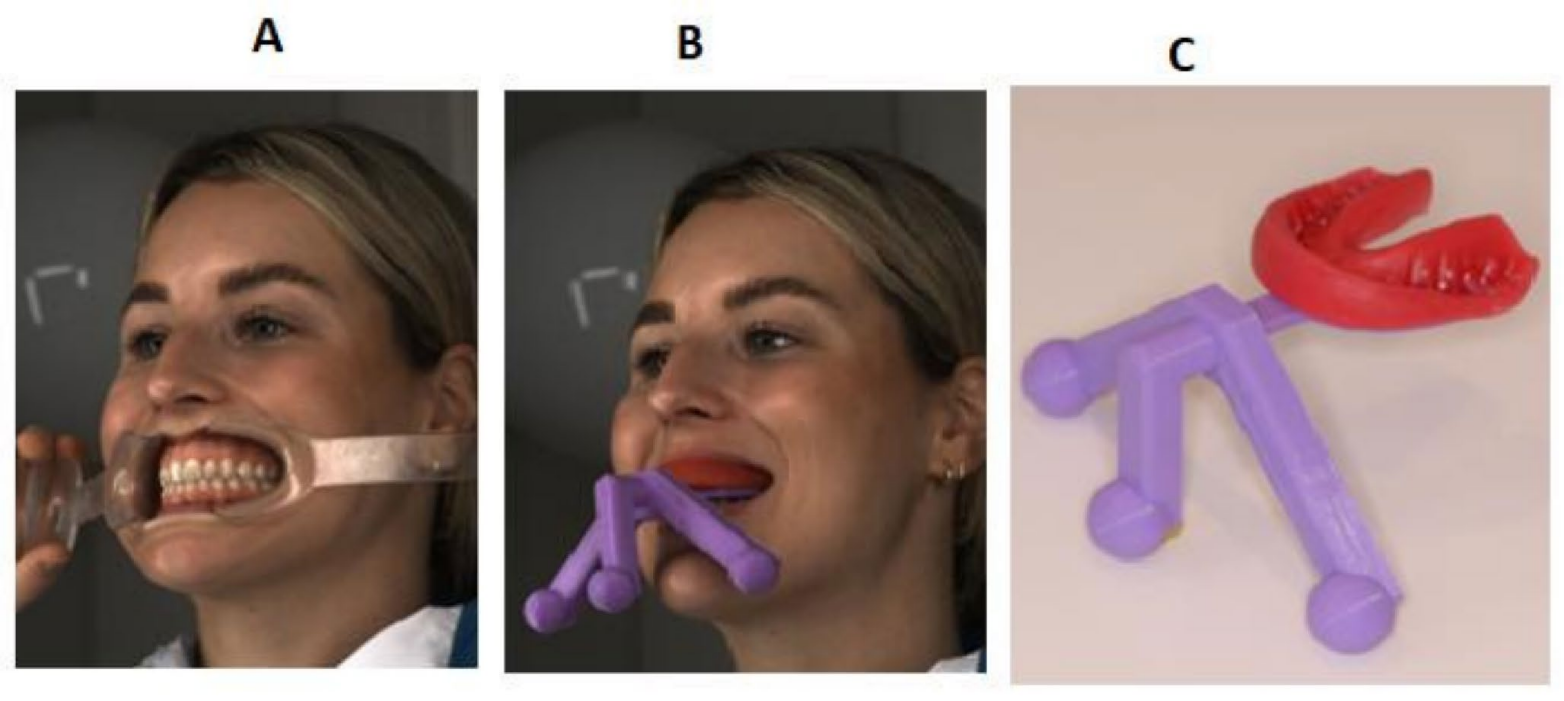
**A**. Method A: 3D stereophotogrammetric image with the patient holding the cheek retractors by herself and pulled backwards as much as possible. **B**. Method B: 3D stereophotogrammetric image with tracer. The wax is softened with warm water, applied on to the bite fork of the tracer and placed in situ by author (RS). Patient bites firmly on the bite fork for a good impression of the upper arch in the wax. **C**. Detailed image of tracer after procedure

This prospective cohort study was performed in full accordance with regulations of the World Medical Association Declaration of Helsinki, and ICH E6 (R2) guidelines for Good Clinical Practice (GCP). Ethical approval of the local Medical Ethics Committee was obtained (METC East-Netherlands, number 2021–13159). For each selected patient, a cross-sectional dataset was collected. This dataset was used to generate 3D fusion models by three different methods: method A using cheek retractors (Fig. 1a), method B using a tracer (Fig. 1b, c) and method C using a full-skull CBCT. The experimental methods A and B were compared to the reference method C.

### Patient selection

All patients were selected from the department of Orthodontics and Craniofacial Biology of the Radboud University Medical Center Nijmegen. Patients referred to our clinic for orthognathic surgery consultation between November 2021 and September 2022 were eligible for inclusion in the study. Patients were asked consecutively to participate in the study when the treatment plan included an orthodontic-surgical treatment. In these cases a full-skull CBCT was obtained, which is the standard of care in our clinic for patients that are scheduled for orthognathic surgery. Therefore, patients were not exposed to extra radiation for this study. There was no predetermined limit on the number of patients included; instead, the sample size was determined by the maximum feasible number of patients available within the timeframe allocated for the data collection. Exclusion criteria were patients with cleft lip and palate and class III patients with a deep reversed front bite. All patients received verbal and written information about the study. Written informed consent was obtained for participating in this study from all patients and/or their legal guardians in case of minors.

### Data acquisition

The following dataset was collected for each patient:

1. An intra-oral scan (TRIOS 3, 3Shape, Copenhagen Denmark).
2. A 3D stereophotogrammetric image (3dMD FaceSystem, 3dMD, London, UK) with the face in rest and the soft tissues in a relaxed position.
3. A 3D stereophotogrammetric image with cheek retractors and the teeth in occlusion.
4. A 3D stereophotogrammetric image with the tracer in situ.
5. A full-skull CBCT scan (KaVo 3D eXam (KaVo, Biberach, Germany + field of view: 16D x 13 H cm; scan time 8.9s; voxel size 0.3 mm; exposure time 3,7s; 120kVp and 5 mA).
6. A CBCT scan of the tracer with the patient’s occlusal impression in the wax (same specifications as the scan mentioned in point 5).

### Processing of data

The digital dataset was used to generate a 3D fusion model for each of the three different methods:

1. Method A: a fusion model of the intra-oral scan and the 3D stereophotogrammetric extra-oral image with cheek retractors. First the 3D stereophotogrammetric image in rest was superimposed on the 3D stereophotogrammetric image with cheek retractors. Because of the deformity of the lower half of the face using cheek retractors, the forehead was used as matching area for the automatic superimposition. Second, the intra-oral scan was superimposed on the 3D stereophotogrammetric image with cheek retractors. The visible surfaces of the teeth were used as matching area for the fusion.
2. Method B: a fusion model of the intra-oral scan and the 3D stereophotogrammetric extra-oral image with the tracer. First the 3D stereophotogrammetric image in rest was superimposed on the 3D stereophotogrammetric image with the tracer with again the forehead as matching area. Second, the digital image of the tracer with the wax-bite was superimposed on the 3D stereophotogrammetric image with the tracer. The tracer was used as a matching area for this part of the fusion. Finally, the occlusal surfaces of the intra-oral scan were superimposed on the occlusal impressions in the wax-bite of the tracer.
3. Method C: a fusion model of the intra-oral scan, the 3D stereophotogrammetric extra-oral image of the face in rest and the full-skull CBCT scan. First the superimposition of the intra-oral scan on the CBCT image was performed with IPS CaseDesigner^®^, as this is a reliable software package for the fusion of the intra-oral scan on the CBCT image [23]. Second the 3D stereophotogrammetric image of the face in rest was superimposed on the soft tissue model of the CBCT. This part of the fusion was matched on the soft tissue surface of the forehead and the zygomatic arch area, since the CBCT only displays the lower part of the forehead which is insufficient for a proper superimposition.

**Fig. 2:**
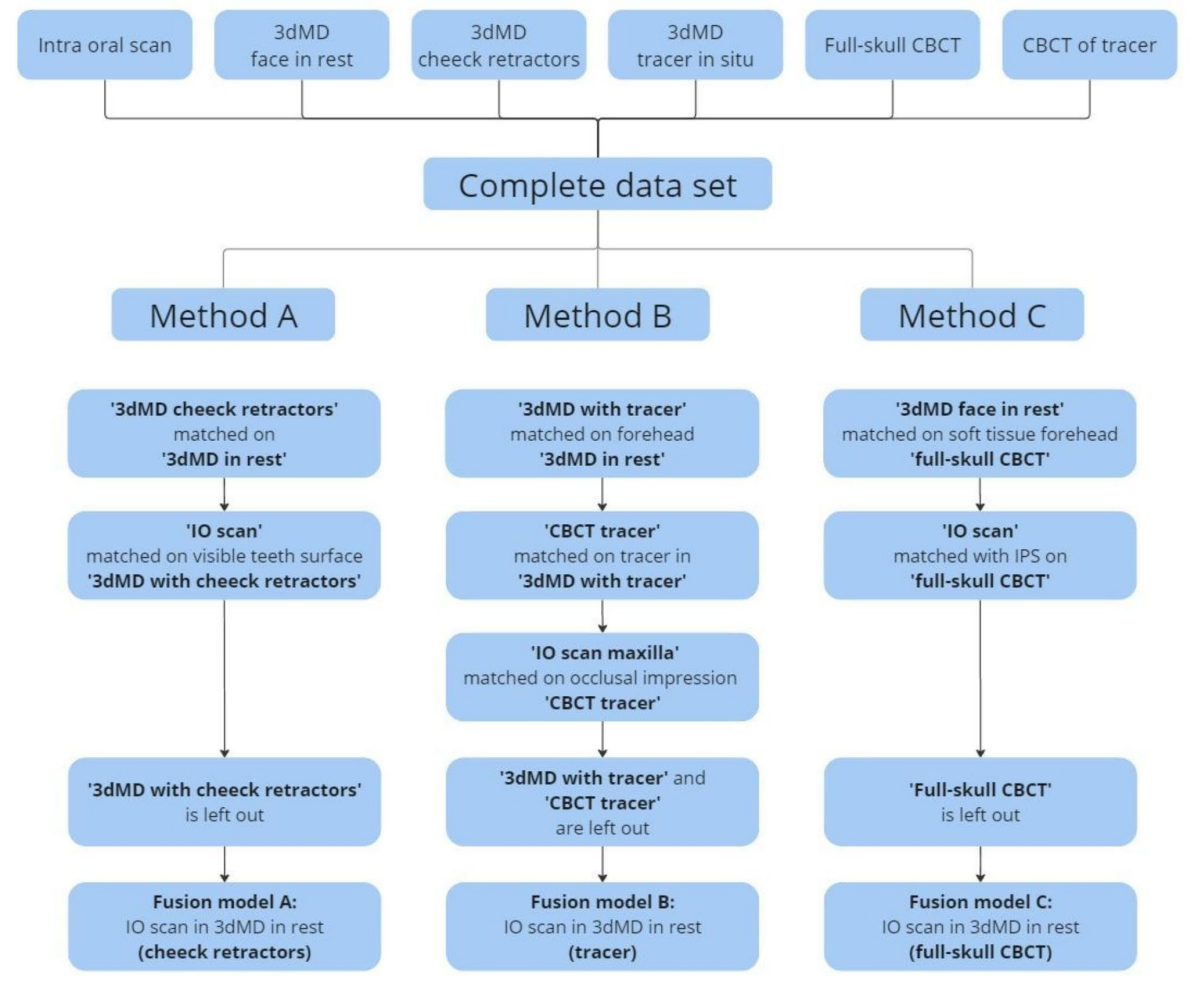
Workflow chart for the workflow of the two experimental (methods A and B) and for the gold standard (method C)

**Fig. 3:**
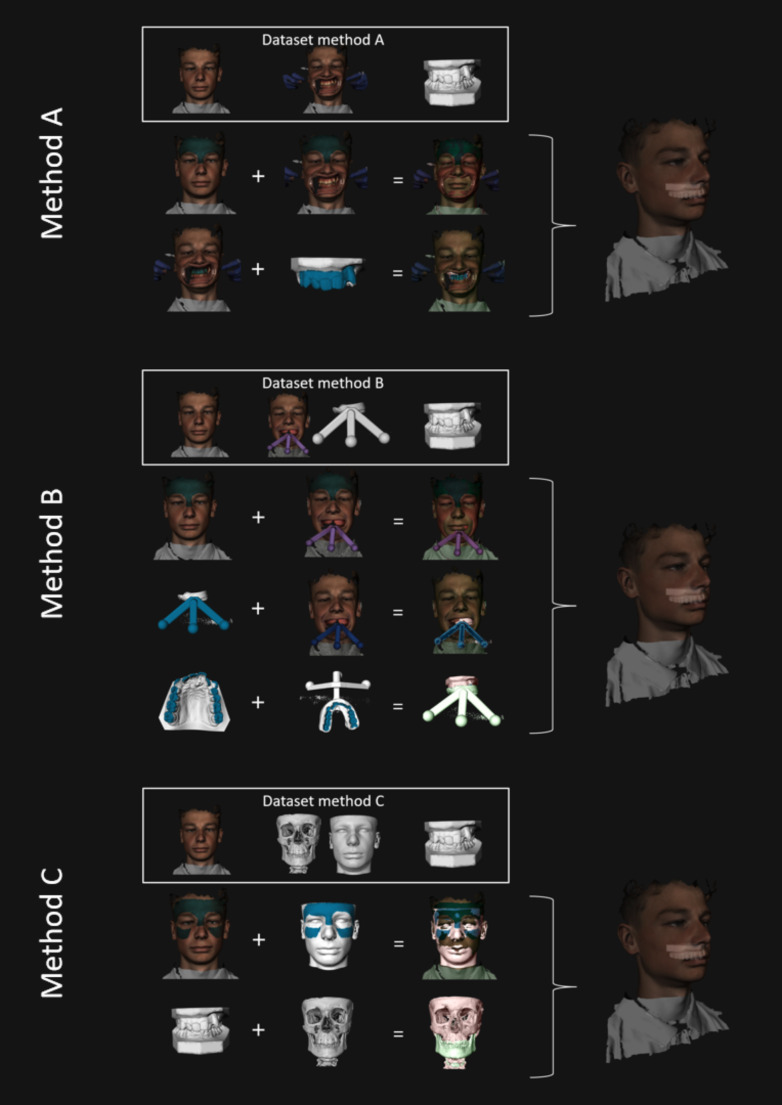
Iconographic description for the workflow of the two experimental (methods A and B) and for the gold standard (method C)

The fusion of all digital files (excluding the superimposition of the IO scan into the CBCT for fusion C which was done with IPS CaseDesigner^®^) were conducted with the 3D software program 3DMedX^®^ (v1.3.0.0, 3DLab Radboudumc, Nijmegen), by two authors (RS and MC). Fusion model C was used as the gold standard reference model for experimental models A and B. All fusion workflows are displayed in Figs. 2 and 3.

### Outcome measurements

**Fig. 4:**
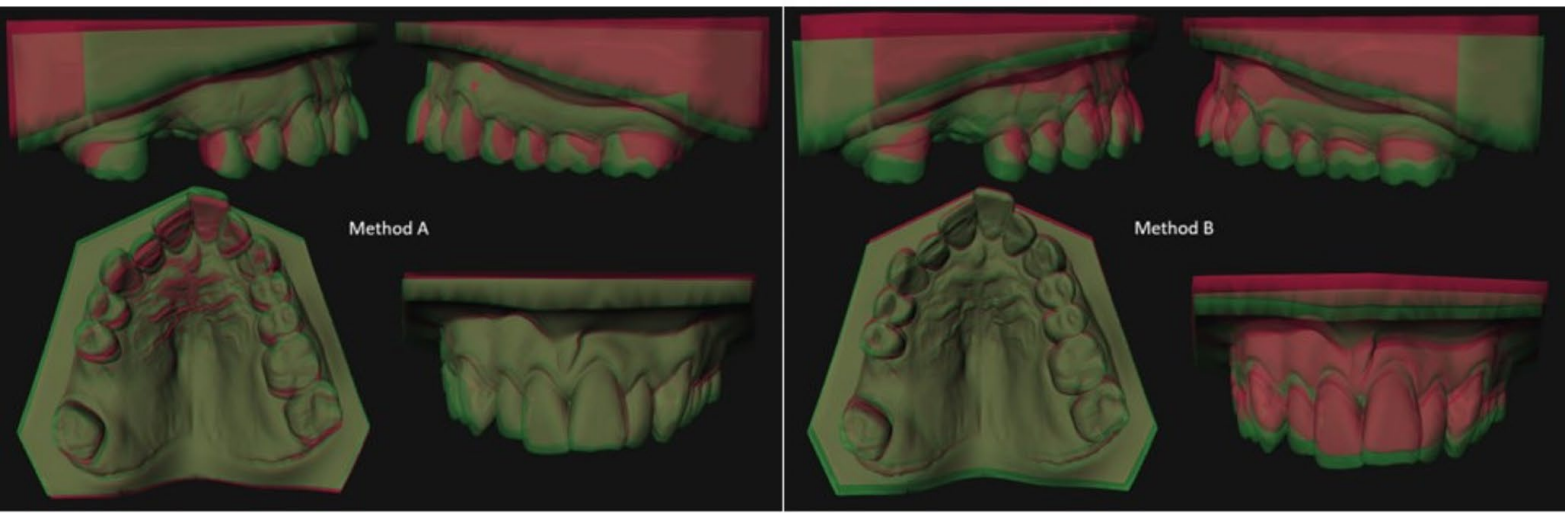
Visualization of the accuracy for method A(left) and method B(right). The experimental models A and B are shown in green and the reference model C in red

The accuracy of the experimental methods was assessed by comparing the position of the maxilla in the experimental models A and B with the position of the maxilla in reference model C (Fig. 4). The exact differences between the methods were calculated by subtracting the position of the maxilla in fusion model A minus the position in fusion model C, the position in model B minus the position in model C and the position in model A minus the position in model B. The center of the STL file representing the maxilla was automatically marked as measuring point and used to asses translation and rotation of the whole maxilla. These measurements were done for six degrees of freedom and defined in translation for X(transversal), Y(vertical) and Z(sagittal) and in rotation for P(pitch), R(roll) and Y(yaw) (3DMedX^®^). These results were imported into Microsoft Excel (Microsoft 365 MSO, version 2308). All measurements with a negative value were then converted into positive values. Finally, the euclidean distance was calculated based on the differences in translation between the models using the formula: √(ΔX)^2^ + (ΔY)^2^ + (ΔZ)^2^.

### Intra- and interobserver agreement

All fusions and measurements in 3DMedX^®^ were done by one author (RS). The fusions and measurements of all 18 subjects were redone after approximately 8–10 weeks by the same author to assess the intra-observer agreement. An other author (MC) redid the fusions and measurements of 9 randomly selected subjects to assess the inter-observer agreement.

### Statistical analysis

All tests between both methods comparing the deviation of A with the deviation of B were performed by applying paired t-tests. For the intra and inter observer analyses, the difference in deviation again was analysed with paired t-tests. In addition to that, the reliability was calculated as the Pearson correlation between both measurement series. The duplicate measurement error (DME) was calculated as the standard deviation of the difference between the measurement pairs, divided by √2.

## Results

Initially nineteen patients were included in this study, but due to lack of matching surface on the forehead one patient had to be excluded. Therefore, a group of eighteen patients, nine men and nine women, aged 16 to 59 years, were included in this study.

### Inter- and intra-observer agreement

For the inter-observer agreement, in one out of fourteen comparisons a statistically significant difference between observers was found (Table 1). Specifically, for the pitch in method B the deviation is 0.247 degrees (95% CI [0.007 … 0.486], *p* = 0.045). For the intra-observer agreement, in six out of fourteen comparisons a statistically significant difference between observers was found (Table 2). For these six measurements the maximum deviation is 0.346 mm or degrees (95% CI [ 0.099 …0.593], *p* = 0.009). The reliability was greater than 0.6 for twenty out of twenty eight measurements, while eight of the twenty eight measurements had a reliability lower than 0.6 due to some outliers. Measurements with a low reliability showed no statistically significant changes in inter- and intra-observer assessments, whereas measurements with high reliability demonstrated statistically significant changes.

**Table 1:** Results of the inter-observer assessment for method A compared to method C and method B compared to method C

Inter-observer agreement					
Method A	Reliability	DME	diff	95% CI	*p*
X	0.923	0.198	0.152	[-0.063 … 0.367]	0.142
Y	0.609	0.636	0.189	[-0.502 … 0.881]	0.545
Z	0.839	0.372	0.348	[-0.056 … 0.752]	0.082
Euclidean distance	0.533	0.383	-0.054	[-0.470 … 0.361]	0.770
R	0.846	0.569	-0.242	[-0.860 … 0.376]	0.392
P	0.576	1.112	-0.659	[-1.869 … 0.550]	0.244
Ya	0.200	1.352	0.532	[-0.938 … 2.002]	0.428
**Method B**					
X	0.795	0.463	-0.190	[-0.693 … 0.314]	0.410
Y	0.974	0.200	0.167	[-0.051 … 0.385]	0.115
Z	0.934	0.300	0.023	[-0.303 … 0.348]	0.877
Euclidean distance	0.977	0.171	-0.090	[-0.276 … 0.097]	0.299
R	-0.125	1.254	0.511	[-0.852 … 1.875]	0.412
P	0.978	0.220	0.247	[ 0.007 … 0.486]	0.045*
Ya	0.919	0.329	0.020	[-0.337 … 0.378]	0.898

**Table 2:** Results of the intra-observer assessment for method A compared to method C and method B compared to method C

Intra-observer agreement					
Method A	Reliability	DME	diff	95% CI	*p*
X	0.842	0.276	0.022	[-0.172 … 0.216]	0.813
Y	0.786	0.392	-0.008	[-0.284 … 0.268]	0.952
Z	0.771	0.317	0.254	[ 0.032 … 0.477]	0.028*
Euclidean distance	0.812	0.245	0.160	[-0.012 … 0.332]	0.066
R	0.411	0.756	-0.276	[-0.808 … 0.256]	0.289
P	0.324	1.926	-0.483	[-1.838 … 0.871]	0.462
Ya	0.598	0.704	0.118	[-0.377 … 0.612]	0.622
**Method B**					
X	0.771	0.417	-0.312	[-0.606 …-0.019]	0.038*
Y	0.981	0.351	0.346	[ 0.099 … 0.593]	0.009*
Z	0.943	0.297	0.224	[ 0.015 … 0.433]	0.037*
Euclidean distance	0.972	0.383	-0.324	[-0.594 …-0.055]	0.021*
R	-0.038	0.990	0.524	[-0.172 … 1.220]	0.131
P	0.962	0.289	0.234	[ 0.031 … 0.438]	0.027*
Ya	0.741	0.541	0.094	[-0.287 … 0.474]	0.610

### Accuracy of translation

The euclidean distance of method A showed a significant lower deviation (-0.74, 95% CI [-1.304 … -0.175], *p* = 0.013) than method B. For translation the highest deviation for method A was along the Z-axis (1.15 mm, 95% CI [0.835 … 1.456]). On average this was 0.41 mm (95% CI [-0.02 … 0.839], *p* = 0.061) more than the translation for method B. For method B the translation along the Y-axis gave the highest deviation (1.85 mm, 95% CI [1.224 … 2.467]). On average this was 1.23 mm (95% CI [-1.854 … -0.599], *p* = 0.001) more than method A. Tables 3 and 4 show these results.

### Accuracy of rotation

For both methods A and B the pitch gave the highest deviation, respectively 2.51 degrees (95% CI [1.756 … 3.272]) and 1.14 degrees (95% CI [0.679 … 1.594]). However, the average pitch for method A was 1.38 degrees (95% CI [0.627 … 2.128], *p* = 0.001) more than the pitch for method B. Tables 3 and 4 show these results.

**Table 3:** The mean for the deviation in each degree of freedom and the euclidean distance. Method A compared to method C, method B compared to method C and method A compared to method B

	Method A		Method B		Method A - Method B		
	mean	CI	mean	CI	mean	CI	*p*-value
X	0.56	[0.369 … 0.759]	0.70	[0.392 … 1.007]	-0.14	[-0.464 … 0.192]	0.395
Y	0.62	[0.353 … 0.884]	1.85	[1.224 … 2.467]	-1.23	[-1.854 … -0.599]	0.001*
Z	1.15	[0.835 … 1.456]	0.74	[0.403 … 1.071]	0.41	[-0.020 … 0.839]	0.061
Euclidean distance	1.57	[1.276 … 1.865]	2.31	[1.712 … 2.907]	-0.74	[-1.304 …-0.175]	0.013*
R	0.85	[0.479 … 1.223]	0.74	[0.189 … 1.291]	0.11	[-0.562 … 0.784]	0.732
P	2.51	[1.756 … 3.272]	1.14	[0.679 … 1.594]	1.38	[ 0.627 … 2.128]	0.001*
Ya	0.88	[0.486 … 1.268]	1.06	[0.660 … 1.465]	-0.19	[-0.748 … 0.378]	0.497

**Table 4:** Number of subjects with the specific value under 2 mm/deg, between 2–3 mm/deg and above 3 mm/deg for method A compared to method C and method B compared to method C

	Method A			Method B		
	X ≤ 2	*2 < X ≤ 3*	*X > 3*	X ≤ 2	*2 < X ≤ 3*	*X > 3*
X	18	0	0	17	1	0
Y	18	0	0	10	5	3
Z	16	2	0	17	1	0
Euclidean distance	13	5	0	7	6	5
R	17	0	1	16	1	1
P	9	5	4	13	5	0
Ya	16	2	0	17	0	1

## Discussion

This study was the first to compare two non-ionizing methods for integrating intra-oral scans into extra-oral 3D stereophotogrammetric images to the gold standard of the CBCT scan. The difference between method A and B for translation along Z- and Y-axis, as well as in roll and yaw rotation, were below 0.5 mm / degrees and statistically non-significant. However, translation along the Y-axis and pitch nshowed statistically significant differences. Method A showed greater accuracy for translation along the Y-axis, while method B was more accurate for pitch.

Several studies have explored accurate and reproducible methods for integrating digital dental casts directly into 3D stereophotogrammetric images without using CBCT [19, 24–26]. These studies consistently concluded that integrating digital dental casts into 3D stereophotogrammetric images without radiation is technically feasible [19, 24–26]. Each study investigated a different integration approach, but unfortunately most of them lacked validation. Rangel et al. and Ritschl et al. did not use CBCT as a reference standard, while Bechtold et al. and Xiao et al. did. Still, none of these studies compared direct integration with integration using a tracer within the same study. Furthermore, all of them used plaster models which have been digitized with a 3D (laser)scanner. Today, however, the intra-oral scanner is widely used and have become the standard for impression taking [27], as they not only facilitate a more contemporary workflow but also support a fully digital process.

Deviations of less than 2 mm or 2 degrees are considered clinically acceptable in 2D cephalometric analyses and orthognathic planning, as reported in the literature [5, 28–31]. In this study, all 2D measurements, except for the pitch in method A (2.51 degrees), fell within this clinically acceptable range. For 3D deviations, the euclidean distance for method A (1.57 mm) was below the clinically acceptable threshold, while the euclidean distance for method B (2.31 mm) exceeded this threshold. Corresponding with the findings of Xiao et al. [24], method A demonstrated the highest deviations in pitch orientation for rotation and in the Z orientation for translation. This can be attributed to the superimposition being primarily based on the visible surfaces of the anterior teeth, with limited or no alignment on the bicuspids. Incorporating a reference point further posteriorly might help reduce these deviations. However, a reference point located further posteriorly cannot be captured when using only cheek retractors.

Method B exhibits lower pitch deviation compared to method A because it incorporates the surfaces of the posterior teeth. However, similar to the findings of Wang et al. [32], method B shows a higher deviation along the (vertical) Y-axis. In this study, this higher deviation may be attributed to the need to convert the tracer with wax bite from CBCT data into a 3D model using a Hounsfield unit threshold. Variations in the threshold value can cause the 3D model to differ in size from the actual tracer with wax bite.

Despite the larger deviations observed in our study, they fall within the same degrees of freedom as those reported in other studies [24, 32]. However, the overall absolute deviations in our research are higher. This could be due to the accumulation of matching errors and/or errors in converting the tracer from CBCT to a 3D model, which may result in deviations of up to 0.30 mm per registration step [33]. Additionally, a design flaw in the tracer could contribute to the larger deviations.

The principle of the tracer with transmission balls used in this study is similar to the one used by Bechtold et al. [25], but is rather different from the tracers used in more recent studies [32, 34]. The larger the distance between the dentition and the tracer’s matching surface, the greater the potential errors due to the lever principle. Another disadvantage of a large tracer is that it couldn’t be digitally captured using a ‘small’ intra-oral scanner, but it needs a large desktop scanner or an additional CBCT-to-3D conversion step, as done in this study. Designing a smaller tracer that is closer to the dentition could improve accuracy. Moreover, adding more detail onto the matching surface of the tracer like Wang et al. [32] could improve the registration target and the matching accuracy with it.

In this study, fusion model C is considered the gold standard, as we use a CBCT as a reference for the fusion of the data. IPS Case Manager, which has been proven to be a reliable method for superimposing CBCT images [23], was used for this process. However, for the construction of this ‘gold standard’ model we need to take into account that it’s a fusion of two consecutive superimpositions, which could introduce minor errors at each step of the fusion process. Though, this method has proven to be a reliable approach for performing these superimpositions [23].

Since the CBCT hardware used in this study does not capture the entire forehead, a proper superimposition of the 3D stereophotogrammetric images onto the soft tissue model from the CBCT for method C could not be achieved. Enlarging the scan to include the forehead would improve the matching but would result in additional radiation exposure. Therefore, we extended the surface area for matching to ensure reliable fusion. To achieve an accurate superimposition, only the most stable region should be used, which is why we selected the zygomatic area for the matching process [21, 35].

One patient had to be excluded due to a forehead being covered by hair. The protocol specified that if the patient’s hair covered the forehead, a hairband should be worn for the capturing of the 3D stereophotogrammetric image. However, the photographer did not use a hairband for this patient, leading to exclusion from the study. As a result, the total sample size was reduced to 18 patients. For future research, it would be favorable to have a larger sample size, including patients with a broader range of orthodontic conditions and demographics. Additionally, performing a sample size and power analysis could be beneficial for future studies.

At a research institute, the time and effort required for non-ionizing methods A and B are manageable. However, this workflow is often too complex and time-consuming for peripheral clinics. While the clinical workflow itself is not the main issue, reducing the time needed for data processing to construct fusion models remains a significant challenge. AI could play a key role in addressing this, as it has already begun to make an impact in modern orthodontic practices and could offer substantial benefits for peripheral clinics [36]. Additionally, the detection of 3D landmarks using automated deep learning algorithms is becoming increasingly efficient, which could further expedite data processing in the future [37]. Another challenge is the accessibility of the 3DMD system used in this study, which is both rather space-consuming and expensive. Nonetheless, more affordable and space-efficient alternatives to the 3DMD system are available on the market [38, 39].

## Conclusion

This study shows promising results for non-ionizing integration methods of intra-oral scans into the 3D stereophotogrammetric images. With improved accuracy in pitch in method A and translation along the Y-axis in method B, all measurements will be within the clinically acceptable threshold. However, since these two measurements exceed the clinically acceptable thresholds, the whole model positioning is less accurate. Therefore, the main goal in further research should be to improve the accuracy of the pitch rotation in method A and the translation along the Y-axis in method B. Therefore, future research should focus on:

- More accurate and detailed 3D stereophotographic imaging of the dentition in method A to achieve more pitch accuracy.
- Designing the most ideal tracer design to minimize shift due to the lever principal and to optimize the surface registration for the 3DMD camera system.
- Developing a workflow with fewer matching steps to reduce the accumulation of matching errors.

Once both methods achieve clinically acceptable accuracy, research could shift towards developing workflows more streamlined and suitable for peripherical clinics. From our point of view, some suggestions are:

- to use AI and automated matching with deep learning to reduce the processing time.
- to investigate the suitability of the affordable and space-efficient alternatives to the 3DMD system.

## Data Availability

Data is provided within the manuscript and supplementary information files. All data underlying this article will be shared on reasonable request to the corresponding author.
